# Biocompatibility and Bioactivity Evaluation of Novel Calcium Silicate-Based Sealer: *In* *Vitro* Study on Human Dental Pulp Stem Cells

**DOI:** 10.1055/s-0045-1802566

**Published:** 2025-05-01

**Authors:** Anggraini Margono, Redho Sara Pratiwi, Anggita Dini Nofarina, Dewa Ayu NPA, Ike Dwi Maharti, Romilda Rosseti

**Affiliations:** 1 Department of Conservative Dentistry, Faculty of Dentistry, Universitas Indonesia, Jakarta, Indonesia

**Keywords:** sealer, calcium silicate, viability, mineral deposition, migration rate, hDPSC

## Abstract

**Objective** This article evaluates the biocompatibility and bioactivity of a novel calcium silicate-based sealer by assessing its impact on the viability, mineral deposition, wound closure, and migration activity of human dental pulp stem cells (hDPSCs).

**Material and Methods** AH Plus and AH Plus Bioceramic were pulverized and sterilized according to International Organization for Standardization 10993-5:2009. The hDPSCs were stored raw materials, reaching 80% confluence after passing stem cell marker tests (CD90 98%, CD105 99.7%, CD73 94%, and LinNeg 0.5%) and were at passage 5 to 6 after serum starvation for 24 hours. The study consisted of four groups: AH Plus at concentrations of 1:1 and 1:4, and AH Plus Bioceramic at concentrations of 1:1 and 1:4. Viability was assessed using the 3-(4,5-dimethylthiazol-2-yl)-2,5-diphenyltetrazolium bromide (MTT) assay by measuring optical density values, while mineral deposition was evaluated through Alizarin red staining and analyzed with ImageJ software. Migration activity was measured by calculating migration speed and wound closure percentage using a scratch assay at 24 and 72 hours, with results analyzed by ImageJ.

**Statistical Analysis** Viability, migration, and wound closure results were analyzed using one-way analysis of variance. Mineral deposition was analyzed descriptively.

**Results** There were significant differences in the viability and mineral deposition of hDPSCs between calcium silicate-based sealers and epoxy resin-based sealers. Calcium silicate-based sealers showed higher viability and better mineral deposition. The migration speed rate of hDPSCs at 24 hours and wound closure at 24 and 72 hours were significantly greater with the novel calcium silicate-based sealer compared with the epoxy resin-based sealer.

**Conclusion** This study suggests that calcium silicate-based sealers offer advantages over traditional epoxy resin-based sealers, demonstrating superior biocompatibility and bioactivity. These properties may lead to improved clinical outcomes, such as faster healing and fewer posttreatment complications. Further research is needed to explore the full potential of these materials in endodontics.

## Introduction

The main components of root canal filling materials are the core material and the sealer. 1 An important factor in the success of endodontic treatment is the creation of an adequate seal that forms a monolithic bond with the dentin of the root canal, preventing the spread of microorganisms into the canal and reducing the risk of reinfection. The sealer plays a crucial role in achieving this outcome. 2 Ideally, root canal sealers should possess properties such as the ability to adhere to root canal walls, antimicrobial activity, biocompatibility, the ability to promote adequate sealing, a fine particle size, an appropriate setting time, insolubility in tissue fluids, and easy removability from the inside of the root canal. 3

AH Plus is still considered the gold standard sealer due to its good adhesion to dentin, biocompatibility, adequate physical properties, and excellent sealing ability. However, previous studies have identified some limitations of AH Plus, such as its large particle size, which makes it more difficult for the sealer to penetrate dentinal tubules in the apical third of the root canal. 4 Additionally, studies have shown that if the sealer is unintentionally extruded into the periapical region through the apical foramen, its cytotoxic effects are typically more pronounced before it sets. 5 Previous study showed that the toxicity caused by sealers significantly contributes to the periapical tissue destruction, which can lead to a less favorable prognosis. 6

This has driven the continuous development of sealer materials in the field of endodontics, with the goal of achieving periapical tissue regeneration. Endodontic regeneration depends on four factors: stem cells, growth factors, scaffolds, and a sterile environment. 7 To support this regeneration process, bioactive and biocompatible materials are needed to promote regeneration of apical tissues. Biocompatibility refers to the ability of a material to function with an appropriate host response in a specific application. On the other hand, bioactivity refers to the ability of a biomaterial to interact with living cells and tissues to induce specific cellular responses, such as the release of ions or other biological substances. 3 The biocompatibility of sealers is linked to the regeneration of periapical tissues. The bioactivity of a material can be evaluated based on its effects on the migration activity, viability, and mineral deposition of human dental pulp stem cells (hDPSCs).

Calcium silicate-based sealers are materials currently under development in the field of endodontics. These sealers offer several advantages over AH Plus sealers, including better penetration ability into dentin tubules, a shorter setting time, and higher bioactivity. 8 The AH Plus Bioceramic Sealer (Dentsply Sirona, United States), a tricalcium silicate-based sealer in a premixed formulation, was recently introduced. This new sealer has demonstrated a shorter setting time, lower solubility, reduced film thickness, and increased radiopacity compared with the traditional AH Plus sealer. 9 In a study by Seo et al, it was reported that calcium silicate-based sealers promote better cell migration of hDPSCs than epoxy resin-based sealers, which showed no cell migration. Additionally, calcium silicate-based sealers were found to enhance cell viability and mineral deposition more effectively than epoxy resin-based sealers. 10 One distinguishing feature of this sealer is the presence of dimethyl sulfoxide as a filler, which is not found in other calcium silicate-based sealers. Additionally, it has a lower proportion of calcium silicate but a higher content of zirconium oxide

Research has shown that hDPSCs have clonogenic potential, high proliferation, and can differentiate into various tissues (odontogenic, osteogenic, and adipogenic) to regenerate tissues. 11 hDPSCs can differentiate into progenitor cells, which requires different times, with osteogenic differentiation taking approximately 14 to 21 days. 12 Ledesma-Martínez et al reported that DPSCs are multipotent cells that can produce mineralized tissues, extracellular matrices, and dentin, pulp, and periodontal ligament structures. 13

Different formulations of calcium silicate-based sealers are being developed to identify the optimal composition for clinical use, which will influence the biocompatibility and bioactivity of each material. However, the impact of a novel calcium silicate-based sealer, such as the AH Plus Bioceramic, on the viability, migration rate, and mineral deposition of hDPSCs has not yet been reported. The purpose of this study was to investigate how this new calcium silicate-based sealer affects the viability, migration rate, and mineral deposition of hDPSCs as an initial step in the regeneration process. This could potentially position it as the latest gold standard in sealer materials.

## Material and Methods

This study's protocol was approved by the Ethical Committee of the Faculty of Dentistry, Universitas Indonesia, Number:13/Ethical Exempted/FKGUI/III/2023 with protocol number, Number 050180223, Number: 05/Ethical Exempted/FKGUI/III/2023 with protocol number 050090223, and conducted at Prodia Stem Cell (ProStem) Laboratory.

### Human Dental Pulp Stem Cells

Samples consist of hDPSCs derived from previous studies. These cells were subjected to serum starvation for 24 hours at a concentration of 5 × 10 ^3^ until they reached confluence at P5-6. The pulp stem cells were then transferred to 96-well plates, with each treatment group containing two samples, and each sample were triplicated. As a result, each treatment group had a total of six samples

## Experimental Disks of Various Root Canal Sealers

The root canal sealers tested in this study were AH Plus Bioceramic (Dentsply) and AH Plus (Dentsply). All experimental sealers were mixed according to the manufacturer's instructions. AH Plus Bioceramic, a premixed sealer, was placed in a disposable syringe. AH Plus sealer was mixed at a 1:1 and 1:4 ratio according to the manufacturer's instructions. Disks of each root canal sealer, 5 mm in diameter and 2 mm in height, were made under aseptic conditions using sterile Teflon molds. All disks were stored in a 100% humidity chamber at 37°C for 48 hours to set. 10

## Biocompatibility

### Cell Viability Assay

Cell viability is tested with MTT assay. As much as 10 μL of pulp cell cultures in each well were added to the MTT assay and incubated at 37°C and 5% CO _2_ for 4 hours. The media were then removed and dimethyl sulfoxide solution (Sigma-Aldrich, Saint Louis, Missouri, United States) (100 μL/well) was added and incubated for 30 minutes at room temperature to dissolve formazan crystals. Subsequently, the absorbance of each well was measured at a wavelength of 570 nm using an enzyme-linked immunosorbent assay reader. The ability of hDPSCs stem cells to survive after 24 hours was observed, and then the test results in the form of hDPSCs absorbance value were converted to cell number percentage.

### Cell Migration and Wound Healing Assay

To evaluate cell migration ability, a scratch wound-healing assay was used. hDPSCs were seeded in 24-well plates (SPL Life Sciences Co., Ltd.) at a density of 1 × 105 cells/well and incubated for 24 hours in a growth medium to allow cell attachment. A scratch was made in the center of the confluent layer of cells using a 20-μL pipette tip. After wounding, cell debris was washed away with phosphate-buffered saline (PBS). To maintain the culture medium above the level of the disk, an additional 1 mL of growth medium was added to each well. Images of the scratch area were taken at 0, 24, and 72 hours using a phase-contrast microscope (Olympus, Tokyo, Japan). ImageJ 1.46r software (National Institutes of Health, Bethesda, Maryland, United States) was used to measure the surface covered by the cells (wound closure) and migration speed rate. The area of cell migration into the scratch area was calculated using the original scratch area as a reference. Each experimental group was analyzed in quadruplicates.

### Alizarin Red Staining Assay

Alizarin Red S (ARS) was used to detect mineral synthesis by hDPSCs. After 21 days, hDPSCs were fixed with 4% paraformaldehyde for 30 minutes and stained with ARS. After staining, the ARS solution was discarded, and the disks were washed with PBS to remove any residual ARS and then mounted. Three wells were tested for each group at the same time point ( *n* = 3 groups). The intensity of the staining was observed under a microscope (Axio Observer, Zeiss, Germany). Wells with cells and no ARS were measured at the same time and used as control.

Microscope images were analyzed using ImageJ software to obtain a descriptive profile of the area density caused by mineral deposition.

### Statistical Analysis

Statistical analysis was performed using one-way analysis of variance (ANOVA) with least significant difference *post hoc* (SPSS 26.0, IBM, United States).

## Results

Based on the one-way ANOVA parametric statistical test, there was a significant difference ( *p* < 0.05) between the treatment groups of calcium silicate-based sealers and epoxy resin-based sealers at concentrations of 1:1 and 1:4 on the viability of hDPSCs at 24-hour observation.

Based on the Bonferroni *post hoc* test in Table 1 , there was a significant difference ( *p* < 0.05) between the group with calcium silicate-based sealer at a concentration of 1:1 and the group with an epoxy resin-based sealer at a concentration of 1:1 at the 24-hour observation.

**Table 1:** Comparison of hDPSC viability test between calcium silicate-based sealers (AH Plus Bioceramic) and epoxy resin-based sealers (AH Plus) with various concentrations at 24-hour observation

Group	AHBio 1:1	AHBio 1:4	AHP 1:1	AHP 1:4
AHBio 1:1		0.000 a	0.000 a	1.000
AHBio 1:4			1.000	1.000
AHP 1:1				0.001 a
AHP 1:4				

Abbreviation: hDPSC, human dental pulp stem cell.

a Bonferoni *post* *hoc* test, *p* < 0.05.

However, there was no significant difference ( *p* > 0.05) between the group with calcium silicate-based sealer at a concentration of 1:4 and the group with an epoxy resin-based sealer at a concentration of 1:4 at the 24-hour observation. Table 1 shows that the highest optical density (OD) values at the 24-hour observation were found in the groups with calcium silicate-based sealer at a concentration of 1:4 and epoxy resin-based sealer at a concentration of 1:1.

**Fig. 1:**
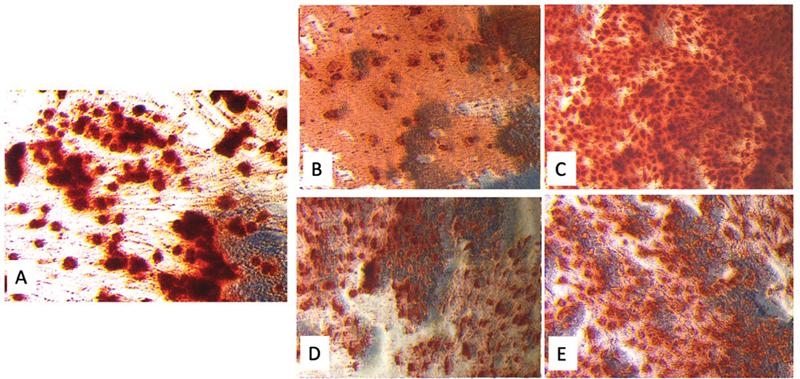
Alizarin red staining after 21 days of observation. ( **A** ) Negative control group (cells + DMEM + osteogenic media). ( **B** ) Calcium silicate-based sealer group with a concentration of 1:1. ( **C** ) Calcium silicate-based sealer group with a concentration of 1:4. ( **D** ) Epoxy resin-based sealer group with a concentration of 1:1. ( **E** ) Epoxy resin-based sealer group with a concentration of 1:4. DMEM, Dulbecco's Modified Eagle Medium.

Bioactivity testing was performed by exposing the sealer to hDPSC on day 21 with a dilution of 1:1 and using ARS, as seen in the microscopic images of mineral deposition on day 21 ( Fig. 1 ).

One-way ANOVA test showed *p* < 0.05, indicating a significant difference in the mineral deposition of hDPSCs between the calcium silicate-based sealers group and the resin-based sealers group at concentrations of 1:1 and 1:4 at 21-day observation.

The measurement of migration speed rate and wound healing analysis was done by a double observer. The interrater reliability test was carried out with an intraclass correlation coefficient of *r* = 0,999 (SPSS 26.0, IBM).

**Fig. 2:**
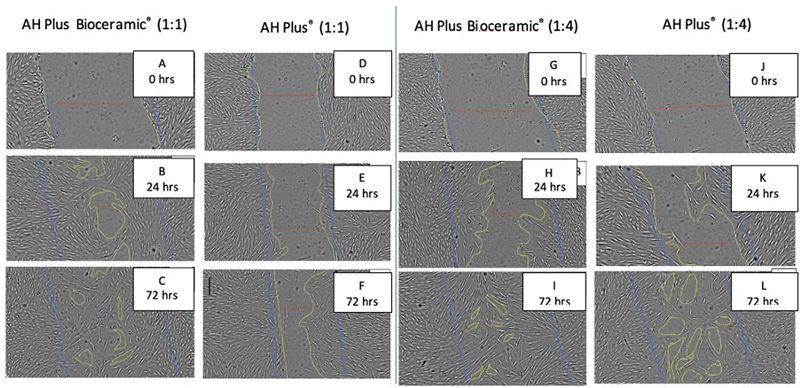
Microscopic and ImageJ views of each treatment group at 0, 24, and 72 hours. Group human dental pulp stem cells (hDPSCs) were treated with AH Plus Bioceramic 1:1 after scratch assay for 0 ( **A** ), 24 ( **B** ), and 72 hours ( **C** ). Group hDPSCs were treated with AH Plus 1:1 after scratch assay for 0 ( **D** ), 24 ( **E** ), and 72 hours ( **F** ). Group hDPSCs were treated with AH Plus Bioceramic 1:4 after scratch assay for 0 ( **G** ), 24 ( **H** ), and 72 hours ( **I** ). Group hDPSCs were treated with AH Plus 1:4 after scratch assay for 0 ( **J** ), 24 ( **K** ), and 72 hours ( **L** ).

In this study, wound closure was measured shortly after scratching (initial wound length: 0 hour), after 24 hours, and after 72 hours (final wound length). Microscopic images ( Fig. 2 ) were obtained during observation and then processed and measured using ImageJ software. (Rasband, W.S., ImageJ, U.S. National Institutes of Health, United States). At a concentration of 1:4, AH Plus Bioceramic exhibits a higher migration rate after 24 hours and optimal wound closure at 72 hours, as depicted in Tables 2 and 3 .

**Table 2:** hDPSC migration speed rate (nm/min) of various sealers with concentrations of 1:1 and 1:4 at 24 and 72 hours

Sealer	24 h (mean ± SD)	72 h (mean ± SD)	*p-* Value
AHBio 1:1	34.21 ± 0.13	16.34 ± 0.04	0.000 b
AHBio 1:4	45.37 ± 0.18	16.47 ± 0.19	
AHP 1:1	18.21 ± 3.48	13.32 ± 1.93	
AHP 1:4	28.22 ± 0.66	15.07 ± 0.29	
*p-* Value	0.0003 a	0.014 a	

Abbreviations: ANOVA, analysis of variance; hDPSC, human dental pulp stem cell; SD, standard deviation.

a One-way ANOVA test, *p* < 0.05.

b Paired *t* -test, *p* < 0.05.

**Table 3:** Comparison of wound closure hDPSCs (%) with various sealer concentrations of 1:1 and 1:4 at 24 and 72 hours of observation time

Sealer	24 h (mean ± SD)	72 h (mean ± SD)	*p-* Value
AHBio 1:1	52.5 ± 9.40	80.22 ± 1.27	0.000 b
AHBio 1:4	34.13 ± 9.20	96.56 ± 2.20	
AHP 1:1	24.16 ± 20.9	58.67 ± 9.69	
AHP 1:4	42.61 ± 15.8	79.20 ± 6.14	
*p-* Value	0.246 a	0.000 a	

Abbreviations: ANOVA, analysis of variance; hDPSC, human dental pulp stem cell; SD, standard deviation.

a One-way ANOVA test, *p* < 0.05.

b Paired *t* -test, *p* < 0.05.

## Discussion

In order to achieve successful endodontic regenerative therapy, materials in the field of endodontics continue to evolve. Currently, the gold standard for endodontic sealers is AH Plus. AH Plus offers several advantages, including low solubility, good dimensional stability with minimal expansion, strong bonding capability with dentin, excellent density, adequate flow properties, and a setting time that meets International Organization for Standardization standards. 14 However, studies have identified limitations with AH Plus, such as mutagenicity, cytotoxicity, hydrophobicity, and an inflammatory response. 15 As a result, a new generation of sealers, known as calcium silicate-based sealers, has recently been developed. These sealers exhibit bioactivity and biocompatibility, supporting the regeneration of periapical tissues. Bioactivity refers to the ability of a biomaterial to induce specific responses in living cells or tissues. 3 Previous studies have identified several parameters that indicate the bioactivity potential of a material, including its ability to induce the formation of carbonated apatite on the material's surface, the formation of mineralization nodules in cells, the development of mineralized tissue *in vivo* , and the bioactivity potential in cells. 16

Calcium silicate-based sealers facilitate the formation of hydroxyapatite, enhancing the bond between dentin and gutta-percha after setting. 16 AH Plus Bioceramic falls within the category of calcium silicate-based sealers. Its composition includes *zirconium dioxide* (50–70%), tricalcium silicate (10–15%) as a bioactive component, dimethyl sulfoxide (10–30%), and lithium carbonate (0.5%) as *thickening agents* . 17 Each component contributes uniquely to the bioactivity of the material. One distinguishing feature of this sealer is the presence of dimethyl sulfoxide as a filler, which is not found in other calcium silicate-based sealers. Additionally, it has a lower proportion of calcium silicate but a higher content of zirconium oxide.

According to Lin et al, zirconium dioxide exhibits excellent biocompatibility creating an environment conducive to regeneration and bone formation, enhancing osteoblast adhesion and proliferation, as well as promoting mineral deposition. 18 Moreover, dimethyl sulfoxide, as a thickening agent, offers advantages such as a high-temperature stability and antibacterial properties. 19

In this study, epoxy resin-based sealers (AH Plus) demonstrated lower viability values than calcium silicate-based sealers. This may be attributed to the release of formaldehyde by epoxy resin-based sealers, which is toxic and can lead to severe inflammatory reactions and delayed healing. 20 These results align with previous study showing that calcium silicate-based sealers positively influence the survival and proliferation of hDPSCs. In a comparison between calcium silicate-based sealers (AH Plus Bioceramic) at a 1:1 concentration and epoxy resin-based sealers (AH Plus) at a 1:1 concentration, AH Plus exhibited better viability values. This could be due to the slower setting time of AH Plus Bioceramic. The final setting of AH Plus Bioceramic is 20 hours, compared with 17 hours for AH Plus, meaning it may continue to release cytotoxic substances before completely setting. 21 Further, the OD values of AH Plus Bioceramic sealer at a 1:4 concentration were higher than at a 1:1 concentration, which aligns with the literature stating that cytotoxicity decreases with lower concentration resulting in better viability values at a 1:4 concentration. The reaction between Ca ^2+^ ions and OH ^-^ ions will produce calcium hydroxide. The presence of calcium hydroxide can cause surface necrosis in the area in contact with the material. In addition, the low viability value of AH Plus Bioceramic with a concentration of 1:1 was due to the ready-to-use form of preparation of the sealer, making the composition inside not hydrated until it comes into direct contact with the extracellular fluid of hDSPC. 22

Alizarin red staining was used to observe mineral deposition in all groups of calcium silicate-based sealers with various concentrations for 21 days. The results of the analysis viewed using the ImageJ software showed that mineral deposition was higher in calcium silicate-based sealers with a concentration of 1:4 compared with epoxy resin-based sealers (AH Plus). This is in line with the increase in cell viability at lower concentrations of calcium silicate-based sealers. 23 24 Calcium silicate-based sealers can induce the formation of hard tissues through the release of calcium ions and the material's hardening reaction. Previous research results have shown the formation of calcite crystals and cellular nucleus mineralization. The addition of zirconium oxide to calcium silicate-based sealers (AH Plus Bioceramic) can reduce inflammatory response, improve biocompatibility, and enhance the bioactive properties of the material. 25 The release of calcium proves that calcium silicate-based sealers possess bioactive properties and have an effect on the differentiation of stem cells and osteoblast progenitor cells. 26

Several studies have reported that the presence of zirconium oxide can enhance the biocompatibility and bioactive properties of materials. Chen et al, in an *in vitro* study, indicated that zirconium ions have the ability to promote the proliferation and differentiation of osteoblast cells. In their study, Alizarin red staining was observed after 21 days of incubation with culture media supplemented with zirconium ions. The study showed an increase in red-stained areas on samples, indicating mineralized bone matrix formation in osteoblast cells after exposure to zirconium ions in the culture medium. 27

Cell migration is a crucial stage in the wound regeneration process. Dental pulp stem cells (DPSCs) can differentiate into various types of tissue cells, supporting tissue healing and regeneration. The migration of DPSC begins from their niche and is controlled by the mitogen-activated protein kinase pathway, activated by calcium hydroxide, which plays a role in proliferation, migration, and differentiation. 28 Calcium silicate-based materials, such as AH Plus Bioceramic, positively affect wound healing by enhancing cell migration and proliferation. Calcium silicate contains components like Ca, P, and Si that influence cell behavior and promote tissue regeneration. Research indicates that the Si ions in this material also support proliferation, angiogenesis, and antibacterial effects. 29

To replicate clinical conditions and reduce cell death, diluting the material was applied in this study. This study utilized a scratch wound -healing assay to evaluate cell migration. 30 31 AH Plus Bioceramic at a concentration of 1:4 promoted a higher migration rate after 24 hours compared with other concentrations ( Table 2 ). Additionally, at 72 hours, AH Plus Bioceramic 1:4 showed the best wound closure results ( Table 3 ).

Calcium silicate-based materials, such as AH Plus Bioceramic, enhance the migration of DPSCs, a critical early phase in tissue regeneration. This material is also believed to increase stem cell viability by promoting more cells to enter the S and G2/M phases, which leads to elevated cell proliferation. The faster migration of these cells results in more rapid wound closure. Thus, calcium silicate-based materials can significantly improve wound healing and tissue regeneration by positively influencing cell migration. 20 24 32

## Conclusion

The study concludes that the novel calcium silicate-based sealer exhibits excellent biocompatibility, as demonstrated by its ability to maintain high hDPSC viability, indicating that it is nontoxic and suitable for endodontic applications. Furthermore, the sealer demonstrates significant bioactivity by enhancing hDPSC migration activity, supporting its potential to facilitate tissue healing and regeneration by promoting cell recruitment to the site of injury or repair. Additionally, the material promotes mineral deposition, which is crucial for dentin and bone formation, underscoring its bioactivity and regenerative potential. These findings suggest that the novel calcium silicate-based sealer is a promising candidate for use in endodontic treatments focused on tissue regeneration and long-term healing outcomes. Further research is warranted to fully explore the potential of these materials in endodontic practice.
